# Evidence for ephemeral middle Eocene to early Oligocene Greenland glacial ice and pan-Arctic sea ice

**DOI:** 10.1038/s41467-018-03180-5

**Published:** 2018-03-12

**Authors:** Aradhna Tripati, Dennis Darby

**Affiliations:** 1 Department of Earth, Planetary, and Space Sciences, Department of Atmospheric and Oceanic Sciences, Institute of the Environment and Sustainability, Institute of Geophysics and Planetary Physics, California Nanosystems Institute, University of California, Los Angeles, CA 90095 USA; 2 European Institute of Marine Sciences (IUEM), Université de Brest, UMR 6538, Domaines Océaniques, Rue Dumont D’Urville, and IFREMER, 29280 Plouzané, France; 3 Department of Ocean, Earth, and Atmospheric Sciences, Old Dominion University, Norfolk, VA 23529 USA

## Abstract

Earth’s modern climate is defined by the presence of ice at both poles, but that ice is now disappearing. Therefore understanding the origin and causes of polar ice stability is more critical than ever. Here we provide novel geochemical data that constrain past dynamics of glacial ice on Greenland and Arctic sea ice. Based on accurate source determinations of individual ice-rafted Fe-oxide grains, we find evidence for episodic glaciation of distinct source regions on Greenland as far-ranging as ~68°N and ~80°N synchronous with ice-rafting from circum-Arctic sources, beginning in the middle Eocene. Glacial intervals broadly coincide with reduced CO_2_, with a potential threshold for glacial ice stability near ~500 p.p.m.v. The middle Eocene represents the Cenozoic onset of a dynamic cryosphere, with ice in both hemispheres during transient glacials and substantial regional climate heterogeneity. A more stable cryosphere developed at the Eocene-Oligocene transition, and is now threatened by anthropogenic emissions.

## Abstract (web summary)

With rapidly disappearing ice, understanding the past behavior of the cryosphere is critical. Here, the authors indicate the initiation and disappearance of glaciation on Greenland and Arctic sea ice coincided in the past, synchronous with Antarctic ice and global ice volume, and a CO_2_ threshold of ~500 p.p.m.v.

## Introduction

One of the most prominent climate changes in our planet’s history involved the transition from early Cenozoic global warmth to the present glaciated state with ice in both hemispheres^1–3^. The timing and cause of this transition is intensely debated^3–5^, largely because of the paucity of sedimentary data from polar regions. Challenging conditions for sample recovery and glacial erosion mean there are few archives of past climate variability available. Studies of the Arctic have focused on a small number of sites and relied upon direct paleoclimate indicators to estimate the onset of ice, including the distribution and chemistry of ice-rafted debris (IRD)^6–16^, and the occurrence of microfossils that live in or near sea ice^15,17^. In addition, oxygen isotope (*δ*^18^O) records for different oceans provide an indirect proxy of ice volume and have been used to determine the timing of ice sheet appearance^3,5,18–22^. Other constraints come from proxy reconstructions of high-latitude temperatures^23–26^.

Based on these techniques, for decades geoscientists have suggested that activity of the cryosphere during the Cenozoic was initiated with the growth of an Antarctic ice sheet ~34 million years ago (Ma), while glacial ice in the Northern Hemisphere formed ~14–6 Ma^1,2,4,6,14,18–20,27–32^. This paradigm argues for two major pulses of Antarctic ice growth, followed by two episodes of Northern Hemisphere ice growth ~20 million years later. The first major glaciation on Antarctica was believed to have occurred near the Eocene-Oligocene boundary, followed by deglaciation, and another major episode of widespread Antarctic ice sheet growth occurred nearly 10 million years later at the Oligocene-Miocene boundary^1,2^. Based on findings of glacially derived sediments on Iceland and in the North Atlantic and Pacific Oceans^6,14,27,33–36^, the onset of ice in the Northern Hemisphere was dated to the late Miocene, with local-scale glaciers developing between 14 and 6 Ma, and an expansion of ice growth to continental-scale glaciation at ~3 Ma.

However, recently some investigations reported evidence for an earlier and more synchronous hemispheric onset of glaciation during the Cenozoic, with the episodic presence of glacial ice^3,5,8,12,13^ in the Northern Hemisphere and Arctic sea ice^13,15–17,37^ as early as the middle Eocene to Oligocene (~47–34 Ma), possibly linked to declining CO_2_^3,13^. Sediments recovered from the tropical Pacific Ocean provided detailed records that support simultaneous, but short-lived, glaciation at both poles during the middle Eocene to early Oligocene^3,5,21^. Specifically, the records show changes in the *δ*^18^O of marine carbonates, including benthic foraminifera and the bulk carbonate fraction of sediments largely comprised of coccoliths. Because carbonate *δ*^18^O is a marker for changes in temperature and water *δ*^18^O, these records reveal synchronous shifts in surface and bottom water hydrography in the tropical Pacific at the same time as changes in the carbonate compensation depth as early as ~44–43^5^ or ~41.5^3^ Ma. Benthic foraminiferal *δ*^18^O records were compared with coeval water-temperature estimates that were derived using Mg/Ca thermometry, a technique standard in Cenozoic studies^22,38–41^, in order to isolate the temperature and seawater *δ*^18^O components of the carbonate *δ*^18^O data^3,5^. Since the growth of ice sequesters ^16^O and leaves seawater more enriched in ^18^O, these results revealed evidence for transient episodes of ice growth beginning in the middle Eocene. Furthermore, using a global isotopic mass balance to calculate ^18^O partitioning between the oceans and ice sheets, these studies inferred the simultaneous presence of ice at both poles, with a minor but significant component of ice storage in parts of the Northern Hemisphere episodically from ~44 Ma^3,5^. However, one study argued the isotopic variations reported in Pacific sediments were not indicative of global ice volume, because such shifts were not observed in Atlantic sediments^4^. Yet it is more likely that *δ*^18^O records differ between the Pacific and Atlantic because of regional water mass effects and/or other localized artifacts (e.g., hiatuses, carbonate diagenesis).

The middle Eocene timing of the initiation of ephemeral Cenozoic bipolar glaciations inferred from Pacific *δ*^18^O records has been supported by more direct evidence, such as the discovery in the Arctic of sea-ice associated diatoms^15,17^ and iceberg-rafted sediment^8,12,13,15,16^. Another piece of evidence has been the study of surface textures of quartz grains recovered from the central Arctic Ocean^13,15,16^, which include dissolution and conchoidal fractures, as both are consistent with transport by sea ice and/or glacial ice. The timing of Arctic IRD and sea ice development was also shown to be broadly consistent with a major decline in atmospheric CO_2_^13^ inferred from one proxy reconstruction derived from boron isotope-based estimates of seawater pH^42^.

More recently, Eocene and Oligocene sediment from the Greenland Sea were shown to have IRD, including quartz grains with various surface textures indicative of ice transport^8,12^. Interpretations of provenance have been limited because surface textures can only provide constraints on transport mechanism(s) and not source regions. Although whole rock element chemistry was used to argue for a possible East Greenland source of IRD between 38 and 30 Ma^8^, this technique is not considered conclusive at establishing provenance in the Arctic and elsewhere due to the impact of variable mineralogy on the elemental chemistry of bulk rock, and the complex and highly variable mineralogy and petrology of Greenland and circum-Arctic rocks and sediments. These previous interpretations are limited by the fact that the method used cannot prove that Greenland was the only possible source for these grains, which is why this method is generally not used in studies of Quaternary ice rafting. Therefore the provenance of IRD to the Greenland Sea and the extent of glacial ice on Greenland remains unknown.

For this study, we apply a well-established chemical fingerprinting technique^9–11,37,43^ to identify specific regions of origin for individual IRD grains. We constrain Greenland glacial ice and Arctic sea ice dynamics during the middle Eocene through early Oligocene. The occurrence of glacial ice on Greenland was synchronous with episodes of circum-Arctic ice-rafting and, in some cases, with proxies for ice volume and carbon cycle changes. Ephemeral glaciations occurred until the earliest Oligocene, marking the end of the transition from early Cenozoic hothouse conditions to a stable icehouse state.

## Results

### IRD source fingerprinting

**Fig. 1:**
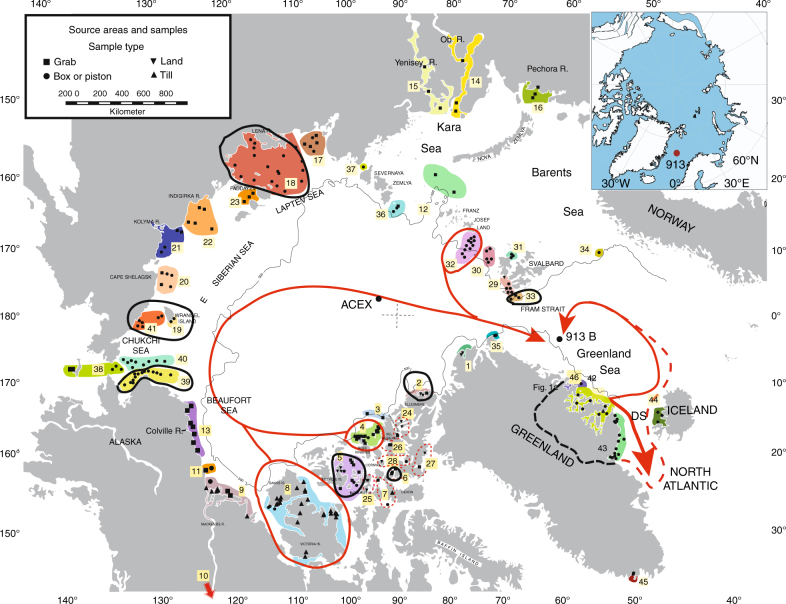
Source areas of ice-rafted debris that drifted to ODP Site 913. Arctic Ocean source areas that contribute significant numbers of Fe oxide grains to any one sample at Site 913 (based on a chemical fingerprinting method^43^) are circled. Red circles indicate more than nine grains; black circles indicate more than 2 grains. Each source area is numbered (note that colors are only for ease of distinguishing one area from an adjacent area). Data on provenance are in Supplementary Data 1–2. The drift tracks are based on modern surface circulation^44,51,52^. The red dashed track is a possible pathway for a small amount of ice^70^. A modeling study indicates the Eocene proto-Greenland Sea would also have been characterized by a cyclonic circulation^47^. DS is Denmark Strait. ACEX indicates location of IODP Arctic Coring Expedition. Black dashed line around source areas #42, #43, and #46 shows approximate extent of ice if it extended to the continental divide. Map adapted from a previous publication^43^. Inset in upper right corner shows paleogeographic map with location of Site 913 in the middle Eocene through early Oligocene^12^. The present location of the site is 75°29.356′ N, 6°56.810′ W, and it is estimated to have moved by less than 0.5° in latitude and longitude during the past 45 million years

We examined 2029 detrital anhydrous Fe oxide grains from the sand fraction of middle Eocene to early Oligocene sediments recovered at Ocean Drilling Program (ODP) Site 913 (Fig. 1), located 360 km east of Greenland. IRD from these sediments have previously been the subject of study^8,12^. In total, we examined 100 sediment samples from Site 913 using the chemical fingerprinting method. Sediment sources were determined for ice-rafted grains that were present in 51 core samples. The remaining 49 samples did not contain Fe oxide grains.

### Sources of oldest IRD

**Fig. 2:**
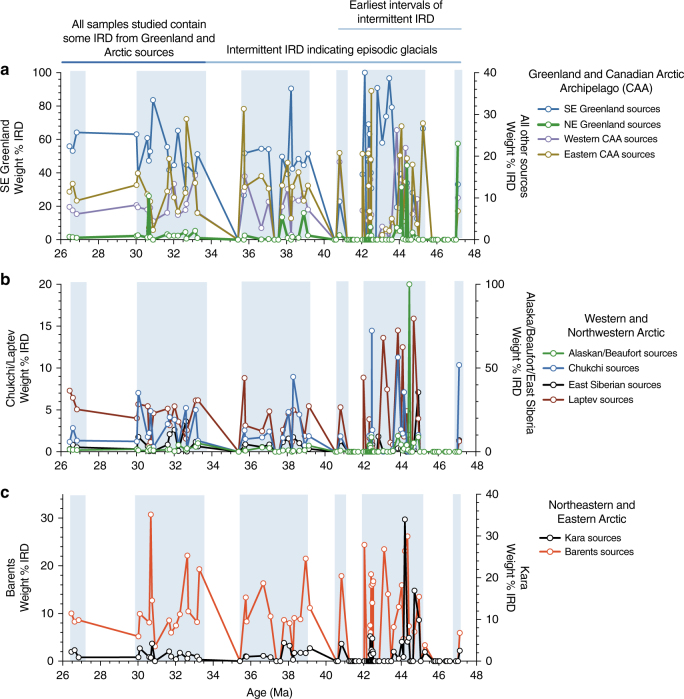
Greenland and Pan-Arctic sources of ice-rafted debris from 48–26 Ma. **a** Greenland and Canadian Arctic Archipelago (CAA) sources; **b** Western and Northwestern Arctic sources; **c** Northeastern and Eastern Arctic sources. Fluctuations in numbers of Fe oxide grains from various sources indicate ephemeral glaciations on Greenland at times when there was sea ice drifting to Site 913 from multiple regions of the Northern, Western, and Southern Arctic. The ice-rafted debris attributable to specific sources is based on chemical fingerprinting of Fe oxide grains. Data are in Supplementary Data 2. NE Greenland = source area (SA) 1, 35 in Fig. 1; SE Greenland = SA 42, 43, and 46; Eastern Canadian Archipelago (CAA) = SA 2–6, 24–28; Western CAA = SA 5, 7–8, 25; Kara = SA 12, 14–16, 36,37; Laptev = SA 17–18, 23; Chukchi = SA 38–41; East Siberian = SA 19–22; Alaskan/Beaufort = SA 9–11, 13; Barents = SA 29–34. Weight % = percent X number of Fe oxide grains matched to a source/10 where 10 is a very conservative estimate of the number of grains that are very significant, in order to avoid high percent values where there are low numbers of Fe oxide grains matched. Zero values indicate a sample did not contain Fe grains from a source in the region. Vertical blue bars mark intervals with samples containing IRD. The Eocene-Oligocene boundary is ~34 Ma

**Fig. 3:**
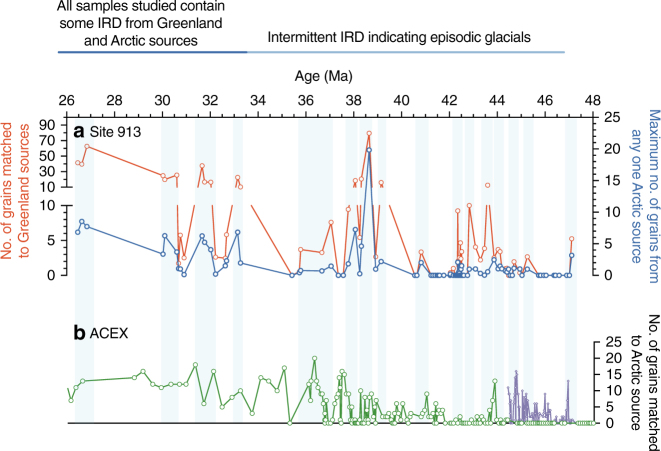
Greenland and circum-Arctic sources of ice-rafted debris from 48–26 Ma delivered to Site 913 in the Greenland Sea and the ACEX site in the central Arctic Ocean. Vertical blue lines indicate intervals with significant numbers of grains matched to a source at Site 913. **a** At Site 913, the number of grains matched to sources on Greenland (red line) and to Arctic sources (blue line). Vertical blue bars mark intervals with samples containing IRD. **b** Published ACEX data^37^ reported using ages calculated using one published age model^69^ (green line) that indicate an Arctic sea ice history corroborated by our results, after factoring in differences in resolution and errors in age models for both sites. Purple line shows the same data but with sample ages calculated using a second age model^68^. The Supplementary Information discusses the published ACEX age models in more detail. The Eocene-–Oligocene boundary is ~34 Ma

We find that beginning in the late middle Eocene, IRD originated from multiple sources on Greenland and the circum-Arctic region (Fig. 2**)**, with the oldest sample (~47.1 Ma) containing grains that can be matched to sources in both of these regions (Fig. 3). No samples examined from below this initial IRD peak contained sand-sized or coarser detrital material and no Fe oxide grains were found. The first highly significant influx of ice-rafted Fe oxide grains (≥5 grains from 1 source) from Greenland indicates the presence of glacial ice at 43.6 Ma, followed by several notable peaks in fluxes through 26 Ma (Fig. 3, Supplementary Data 2).

### Fingerprinting results for Eocene and Oligocene samples

From 47 to 26 Ma, there are some intervals when there are large numbers of Fe oxide grains (up to 75 Fe oxide grains, where 2 grains would be statistically significant) matched to two specific source areas on Greenland (Figs. 2, 3). In addition, although some Eocene samples do not contain any IRD from Greenland, all of the Oligocene samples we examined do (Table 1), a pattern that is consistent with ephemeral glacial ice on Greenland during the middle and late Eocene.

**Table 1:** Key events in Site 913 records from ~47–26 Ma

**Age**	**Event**
47.1 Ma	Oldest sample containing ice-rafted grains from Greenland and circum-Arctic sources
43.6 Ma	High influx of ice-rafted grains from Greenland sources
35.4 Ma	Youngest sample examined that is devoid of ice-rafted grains from Greenland sources
33.3–26.5 Ma	All samples examined contain ice-rafted grains from Greenland sources

The first interval of intermittent IRD is in the middle Eocene (~47.1 Ma) and the amplitude of variability in IRD records increases in the late middle Eocene (after 43.6 Ma). Every sample examined in this study from the early Oligocene (i.e., after the Oi-1 glaciation) contains IRD from some Greenland and Arctic sources

### Specific Greenland source areas

**Fig. 4:**
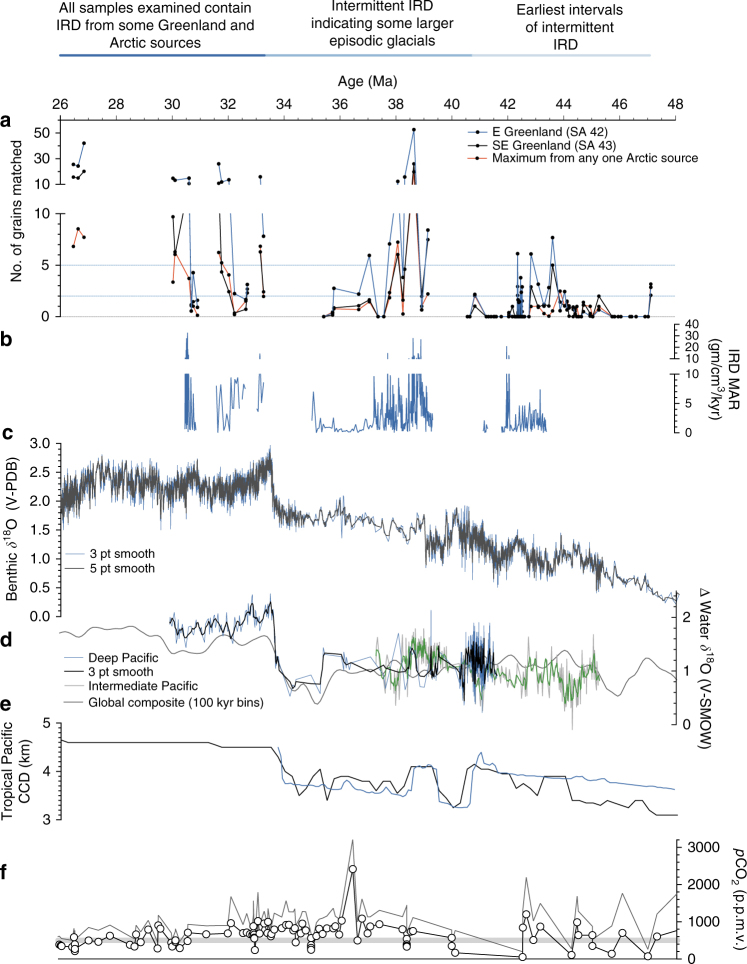
Number of Fe oxide grains from Greenland and Arctic Ocean sources at Site 913 from 48 to 26 Ma compared to proxy indicators of global climate, ice volume, and carbon cycle changes. Supplementary Figures 1–5 show the same figure but with vertical blue lines to mark intervals where Greenland-sourced IRD at Site 913 occurs or when there is an increase in *δ*^18^O, and/or detailed insets of the figure. Comparison shows the occurrence of Greenland ice and circum-Arctic sea ice at Site 913 sometimes, though not always, coincides with increasing benthic foraminiferal *δ*^18^O and Pacific water *δ*^18^O, increases in the carbonate compensation depth, and relatively low *p*CO_2_. **a** Ice-rafted Fe oxide grains from different source regions. Horizontal blue lines indicate 2 and 5 grains matched. **b** IRD mass accumulation rates (MAR) at Site 913 shown, with intervals containing dropstones or grains >250 um in size indicated by underlying dotted blue line. **c** Composite deep-sea benthic foraminiferal *δ*^18^O record is shown with a 3-point and 5-point running mean. **d** Changes in water isotopes at sites in the intermediate and deep Pacific are shown, with 3-point running mean for each, is plotted with a low-resolution global composite of reconstructed water *δ*^18^O. Underlying blue dotted lines indicate intervals where reconstructions show an increase in water *δ*^18^O of more than 0.6 per mil. **e** Carbonate compensation depth (CCD) for equatorial Pacific and tropical Pacific are shown, which are impacted by changes in sea level and carbon cycling. Dotted lines indicating where CCD is relatively deep or increases in depth. **f** Composite proxy *p*CO_2_ reconstruction is shown with lines indicating minimum and maximum values, and gray dotted line marking 500 p.p.m.v. Complete list of data sources in Supplementary Methods

Sources include three regions on East and Southeast Greenland (marked as source areas #42, #43, and #46 in Fig. 1). East Greenland (source area #42 on Fig. 1) is represented by the largest number of ice-rafted Fe oxide grains. The largest contributions from this region are from fjords that contain notable layered intrusives with unique Fe oxide grain compositions. These fjords are known to be sources of large icebergs in recent years^44,45^. Many of the core samples contain an abundance of Fe oxide grains that can be traced to this small source region (Fig. 2). Southeast Greenland (source area #43) is the source of a significant quantity of Fe oxide grains during major episodes of ice-rafting (Fig. 4**;** Supplementary Fig. 1–5). The fluxes from this region mimic those from East Greenland sources (#42 and #46), although the Southeast Greenland values are slightly lower than the two combined East Greenland sources.

### Comparison with published proxy data

Our results were compared with published proxy data, including indicators of global ice volume. We find that several peaks in IRD sourced from Greenland broadly coincide with times of increasing values of benthic foraminiferal *δ*^18^O and/or Pacific water *δ*^18^O (Fig. 4**)**. Some, though not all, of these IRD peaks are also synchronous with changes in carbon cycle indices, including increases in the carbonate compensation depth, and low *p*CO_2_ (<500 p.p.m.v.) (Fig. 4**;** Supplementary Fig. 1–5).

## Discussion

Our chemical fingerprinting approach quantitatively measured 14 elements^43^ in each of 2029 sand-sized detrital Fe oxide grains in sediment samples from the Greenland Sea. The technique enabled us to determine the precise sources of the Fe oxide grains from in the circum-Arctic region and within Greenland. We found that Fe oxide grains could be traced to widespread source areas including the Laptev and Chukchi Seas, the Canadian Arctic Archipelago, and to multiple regions in East and Southeast Greenland (source areas #42, #43, and #46). Sea ice is the likely transport mechanism for grains from many of the circum-Arctic source regions.

However, the probable source of the Fe oxide grains derived from Greenland are icebergs rafted from glacial ice. Glacial transport from Greenland source areas is consistent with the coarse nature of the sediment, the large percentage of quartz grains with evidence of glacial abrasion, and other textural indices^8,12^. Although sea ice may have been important locally along the Greenland coast for entraining sediments, we note the extent of sea ice in the Greenland Sea today is limited to the shelf along most of East Greenland^46^. During the middle Eocene through early Oligocene, when global climate was warmer than today, we assume the extent and thickness of sea ice in this region would have likely been substantially reduced. Modeling studies indicate a cyclonic circulation in the Greenland Sea region for the Eocene^47^, that sea ice was thinner or less extensive than today under these substantially warmer climatic conditions, and that sea-ice drift may have also been significantly faster^48^. The circuitous route required to reach Site 913 from source areas #42, #43, and #46 makes it likely that grains from these Greenland source areas were dominantly transported by icebergs the >400 km distance from the coast to Site 913 (Fig. 1), and not solely by sea ice, given that only icebergs can survive drifting in open water for tens to hundreds of kilometers.

At times in the Eocene and during the Oligocene, numerous large icebergs would have needed to calve from the East and Southeast Greenland source areas simultaneously, given that only a small percentage of icebergs would reach the core site. For the modern surface circulation, ice-drift models suggest that only a small amount of ice would drift east toward Iceland and then north into the Greenland Sea^44,45,49^. This drift track covers more than 1000 km and IRD deposition from icebergs decreases dramatically from calving sites to the last melt-out, similar to the thickness decreases seen at different core sites in Heinrich layers^50^. In order to reach Site 913, most of the icebergs from the Greenland source areas would have drifted south through Denmark Strait^44,45,49,51,52^. Even in the highly unlikely event the East Greenland Current flowed north instead of south in the past, the straight-line drift distance from source area #43 to Site 913 would be over 1000 km. Therefore only a small fraction of icebergs originating in a given source area on Greenland or elsewhere would have survived to deposit IRD at Site 913. It is likely that some of the observed intervals of IRD in cores from Site 913, which can be up to 2–4 cm in thickness, would likely correspond to time intervals of intense glaciation.

During some glacial maxima, regions as far south as 68^o^ latitude, and at least as far north as source area #46 (~73^o^ latitude), were glaciated. The presence of IRD at Site 913 that was simultaneously delivered from source areas #42, #43, and #46 indicates that glacial ice came from widespread areas across East and Southeast Greenland. Fe oxide grains measured in this study matched to every sample cataloged in the database from these three source areas (Fig. 1) and therefore can be attributed to specific locations. Given that other core samples contain no IRD, these results can be best explained if there were some times that were ice free, and during other time intervals there were ephemeral glaciations that were intense enough to cover these areas simultaneously with glacial ice that produced calving icebergs. Although Greenland was not likely to have been completely ice-covered as it is now, our data identify regions of Greenland that were covered with glaciers during parts of the middle Eocene through early Oligocene.

We note the actual distribution of glacial ice at a given time would reflect a combination of summertime temperatures that would largely be controlled by insolation, topography, and greenhouse gas content, as well as moisture availability. Eocene and Oligocene glacial conditions in the Northern Hemisphere would have been affected by the same factors that influence Miocene–Holocene (~24 Ma-present) climate. In addition to temporal variability, there would also have been significant spatial heterogeneity in conditions due to dynamical processes (e.g., factors such as atmospheric and oceanic circulation and heat transport) that today exert a dominant control over regional differences in climate. During transient glacial maxima, if the calving glaciers that supplied icebergs extended inland to the divide, then glacial ice covering these three areas would encompass about 4.3 × 10^5^ km^2^ or ~20% of Greenland (black dashed line–Fig. 1). A caveat that limits a more complete assessment of the full extent of ice coverage is that representation in the database for Greenland is limited, with no samples available from the northeastern regions of Greenland to compare to Site 913 (between source areas #1/35 and #46 on Fig. 1**)**. The abundance of Fe oxide grains in many samples from Site 913 indicates that at times, large numbers of icebergs were produced, consistent with the intermittent occurrence of glaciations, with ephemeral glacial maxima that at times spread far inland and supported calving at the coast (Figs. 1, 2).

Fluctuations in numbers of Fe oxide grains from various source regions in samples from Site 913 indicate that when parts of Greenland experienced ephemeral glaciations, sea ice from multiple regions of the Northern, Western, and Southern Arctic also drifted to the core site. The same sediments containing grains derived from Greenland sources also contain IRD from several circum-Arctic regions, including the Canadian Arctic Archipelago, Western and Northwestern Arctic, and the Northeastern and Eastern Arctic (Figs. 1, 2**)**. These findings are indicative of synchronous ice-rafting in from multiple shelf regions around the Arctic. Several of these sources are represented by significant numbers of Fe oxide grains in Site 913 samples. Sources include Banks and Ellef Ringnes Islands in North America, and Franz Josef Land in Russia (source areas #8, #4, and #32, respectively, in Fig. 1). Many other Arctic source areas are represented by the presence of more than two Fe oxide grains in any one sample (e.g., source areas #2, #5, #18, #19, #33, and #39 in Fig. 1). Some of these areas were probably never glaciated given the lack of glacial deposits, their locations and elevations and thus it is likely that at least some of these Arctic Fe oxide grains were transported by sea ice.

We note that alkenone-based sea surface temperatures derived for the ACEX core from the central Arctic Ocean have been interpreted to reflect summer temperatures of between 6–20 °C during the middle Eocene (~47–38 Ma)^23^. One way to reconcile these two types of data sets, assuming the temperature estimates are robust for this region and time interval, is through geographic and/or temporal variability in conditions. This would require ACEX alkenone data and Site 913 IRD peaks to truly be coeval, and for sea ice to have drifted at a much faster rate, given warmer ocean temperatures and thinner ice^48^. Average drift rates would need to be as high as 25 cm/s for some sources near Banks Island if the drift were along similar trajectories as today; such rates are more than twice what models predict for near ice-free conditions^48^. Alternatively, it is also feasible that individual data points in different proxy records from both sites capture different times within orbital cycles or higher-frequency fluctuations, and together, the data sets provide a glimpse into climate variability during the middle Eocene (i.e., with times of glacial ice on Greenland, sea ice in the circum-Arctic, and ice-free conditions represented in the different data sets), our slightly favored explanation, given that the ocean-climate-cryosphere system would have exhibited variability over a range of timescales including millennial and orbital-scale during the Eocene and Oligocene, just as it has throughout Earth’s history. Another possibility is that directly correlating our data from Site 913 to the published records from ACEX is hampered by limitations on age control. These issues can only be addressed through the recovery and study of more complete sedimentary successions from different regions during future research expeditions.

The provenance data for Site 913 pinpoints the origins of IRD in the Greenland Sea to source regions in East Greenland, Southeast Greenland, and the entire circum-Arctic Ocean, which episodically contributed IRD in the middle Eocene and became more stable by the early Oligocene (~34 Ma, Fig. 2). The coincident peaks in our records from these various source regions indicate that episodic glaciation in Greenland was synchronous with periods of ice-rafting from multiple regions around the Arctic Ocean. Therefore the cold conditions necessary for glaciers to develop on Greenland during these intervals also extended to some, if not all, of the terrestrial areas around the Arctic Ocean. A possible analog for the warm glacial regime on Greenland may include present-day British Columbia and Southeastern Alaska, where there are glacial accumulation areas at high elevation with glaciers extending to and calving at sea level, neither of which has perennial sea ice today.

The large variations in IRD accumulation rate and the contribution of IRD from different sources imply that despite it being reduced in volume compared to the Southern Hemisphere cryosphere, the Northern Hemisphere cryosphere was highly dynamic. The large variability in fluxes from Greenland and circum-Arctic sources, with anywhere from 75 to 0 grains matched in a sample, imply a substantially earlier occurrence of ice accumulation on Greenland than the widely accepted timing of 14 to 6 Ma. Because the oldest samples of Greenland-derived IRD from Site 913 date to ~47 Ma, it is likely glacial ice occurred ephemerally on Greenland beginning as early as the middle Eocene. Not all of the middle and late Eocene (~47–34 Ma) samples we examined contain IRD, indicating that at times ice may have disappeared completely (Fig. 4). In some regions, it is possible that a large ice cap or a small ice sheet was only present during cold summer orbits and was initiated in areas of high elevation, with conditions therefore also being conducive to the development of Arctic sea ice. These results are consistent with the presence of an ephemeral, highly dynamic cryosphere in the Northern Hemisphere beginning in the middle Eocene. On Greenland, episodic glaciations in multiple regions provided adequate ice in the form of icebergs to reach Site 913 during intervals of the Eocene and stabilized by the Oligocene. Specifically, every sample we examined from the early Oligocene contains IRD sourced from Greenland, implying that during and after the Oi-1 glaciation (~33.8–33.6 Ma), glacial ice on Greenland was likely more stable and less ephemeral **(**Fig. 4).

**Fig. 5:**
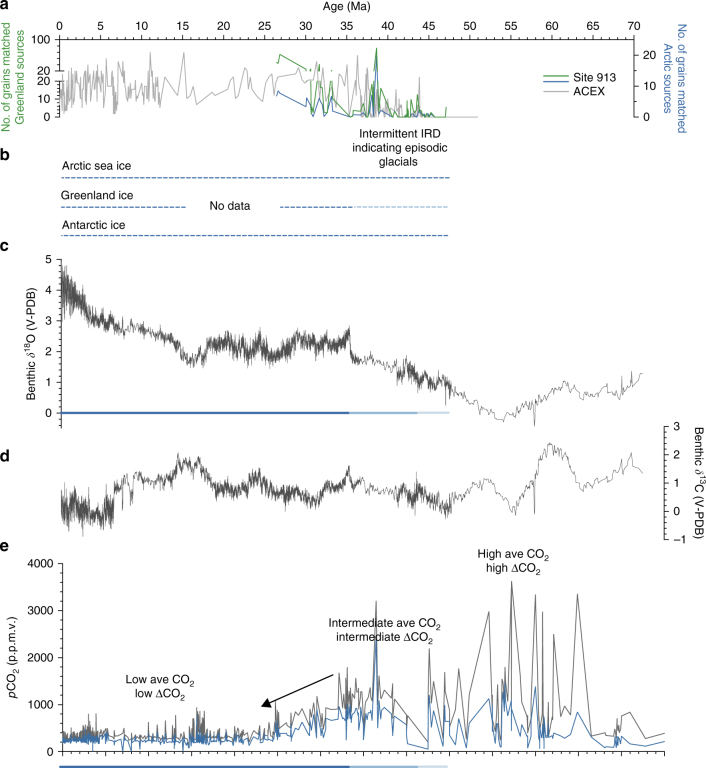
Records of cryosphere and carbon cycle evolution over the past 70 Ma indicate near-sychronous initiation of Arctic sea ice, glaciation on Greenland, and Antarctic glaciation beginning in the middle Eocene (light blue horizontal bars; ~47–44 Ma). Prior to this time, proxy data support high and variable *p*CO_2_, and there is no evidence for IRD. After this time, intermittent IRD appears indicating episodic glacials, synchronous with times of increasing *δ*^18^O. After ~34 Ma, corresponding to the Oi-1 benthic *δ*^18^O excursion, every sample at Site 913 examined in this study contains IRD sourced from Greenland, indicating more stable glacial conditions (dark blue horizontal bars). *p*CO_2_ estimates indicate this event is followed by a reduction to the stable, low mean values that characterize the past ~30 million years. There is generally a correspondence of Fe oxide grain peaks, high *δ*^18^O, and low *p*CO_2_. **a** IRD provenance data from this study for Site 913 indicates grains sourced from Greenland (green) and Arctic sources (blue line). Data for the ACEX core indicates Arctic sources^37^. **b** Blue dotted line indicates times of sea ice in the Arctic Ocean, based on IRD provenance data from this study for Site 913 and record of Arctic sea ice diatoms and IRD in the ACEX core^13,15–17,37^ (ages calculated using one published age model^69^). Underlying dotted blue line based on evidence for glacial ice on Greenland from this study and other IRD studies^6–8,12,14,27,29^. Underlying dotted blue line indicates evidence for glacial ice on Antarctica from two or more proxies (e.g., studies of IRD, sequence stratigraphic record, and/or isotopic data)^1,18,19,21,30,53–55,59^. Color of dashed blue horizontal line reflects inferred glacial frequency and intensity as inferred from proxies. **c**, **d** Compilation of benthic *δ*^18^O and *δ*^13^C data (5-point running mean) with notable isotopic excursions labeled. Intensity of blue horizontal line indicates glacial frequency and intensity as inferred from proxies. **e** Compilation of proxy atmospheric *p*CO_2_ reconstructions (showing minimum and maximum estimates). Color of blue horizontal line reflects inferred glacial frequency and intensity as inferred from proxies. Complete list of data sources in Supplementary Methods

Some of the peaks in the Fe oxide grain data from Site 913 are contemporaneous with known major isotopic excursions in the marine sedimentary record^3,22,53–55^ (Fig. 4). For example, several of the maxima in grains from Greenland and circum-Arctic sources delivered to this site (indicated by blue lines in Fig. 4) are contemporaneous with changes in published records including positive excursions in the Pacific *δ*^18^O record^3,5,21^ which largely reflects temperature and Antarctic ice volume, *p*CO_2_^56,57^, and variations in carbonate sedimentation that reflect changes in sea level and carbon cycling^3,58^ (Fig. 4**;** Supplementary Fig. 1–5). These times are also periods when some records show notable changes in Southern Ocean surface water hydrography and there are reports of IRD derived from Antarctica^59–61^ (Fig. 5). Several of the maxima at Site 913 also broadly line up with changes in published IRD records from the central Arctic^13,15,16,37^, although there are differences in sampling resolution, coring gaps, and age model uncertainties (Fig. 3).

Ultimately, our results are consistent with episodic bipolar glaciation and circum-Arctic ice-rafting beginning in the middle Eocene and throughout the early Oligocene. Significantly, results suggest a broadly concurrent Cenozoic onset and history of Arctic sea ice, glacial ice on Greenland, and glacial ice on Antarctica (Fig. 5**)**. Determining the timing and extent of glaciations in the Northern Hemisphere can help to constrain the tectonic and environmental drivers of climate change^2^. Some have argued that tectonically forced changes in ocean currents circumscribing Antarctica triggered glaciation during the Eocene and Oligocene^62,63^, while a later onset of Northern Hemisphere glaciation occurred largely because of current-driven heat transport changes resulting from the emergence of the isthmus of Panama^64^. An earlier timing for the onset of ice in the Northern Hemisphere indicates ocean heat transport was not likely the sole or direct trigger for the Cenozoic initiation of the first glaciations on Greenland, although the oceanic meridional overturning circulation may have served as a feedback mechanism for ice growth and/or a driver of carbon cycle changes.

Others have argued that greenhouse gas levels may have had a role in driving these transitions^2,3,13,65^, in part based on the timing of ice growth relative to reconstructions of CO_2_ fluctuations. During the Cenozoic, falling *p*CO_2_ may have resulted in temperatures at both poles dropping below critical thresholds for the formation of sea ice and glacial ice^3,13^. Some studies have hypothesized the polar regions in both hemispheres had relatively similar CO_2_ thresholds for the onset of glaciation^3,13^. Alternatively, substantially different CO_2_ and temperature thresholds may exist for glaciation on Greenland and Antarctica due to differences in land mass, latitude, and solar insolation^66^. Our data allow us not only to test whether there is evidence for CO_2_ serving as a critical component of the climate system governing cryospheric evolution during the Cenozoic, but also to evaluate these differing views of thresholds for glaciations.

The IRD source fingerprinting results coincide at times with fluctuations reported in reconstructions of *p*CO_2_ derived from multiple marine and terrestrial proxies (Fig. 4**;** Supplementary Fig. 1–5). Both types of *p*CO_2_ reconstructions show changes during the middle Eocene through early Oligocene. Some of the changes observed suggest that at times, maxima in IRD sourced from Greenland coincided with CO_2_ minima. Comparison with records constraining Northern and Southern Hemisphere polar glacial onset indicates that during multiple intervals, when reconstructed *p*CO_2_ values dropped below ~500 p.p.m.v., there is evidence for glacial ice in both hemispheres (Figs. 4, 5**)**. Thus, our results may support an occurrence of glaciations with broadly similar CO_2_-equivalent and temperature thresholds in both hemispheres^3,13^.

Other factors that link glaciation to CO_2_ include insolation and lapse rates. For example, during intervals with relatively low CO_2_ values and orbital parameters favoring cold polar summers, given thermodynamically constrained values for lapse rates, these times would have been associated with precipitation falling as snow at elevation in the surrounding region, including Greenland, leading to the presence of glacial ice. Figure 5 shows proxy CO_2_ values that provide a dynamically plausible mechanism for Northern Hemisphere ice during the hothouse-icehouse climate transition of the early Cenozoic.

Significantly, the source fingerprinting method provides evidence that the initiation of glacial ice on Greenland, with a possible large ice cap or even small ice sheet, occurred earlier than previously suggested, and that the occurrence of glacial ice in the middle Eocene to early Oligocene was synchronous with pan-Arctic sea ice. These new observations allow us to critically reevaluate our prior understanding, suggesting that previous theories were only working hypotheses and allowing us to work towards a more complete interpretation. In fact, the Cenozoic initiation of glacial ice on Greenland, and sea ice in the Arctic, was close in timing to the development of Antarctic ice. We find evidence for ephemeral glacial ice and sea ice in the Northern Hemisphere, and times of ice-free conditions, until the Oi-1 glaciation, and for more stable glacial conditions in the early Oligocene. Future studies will provide insights into the history of Northern Hemisphere ice and bipolar glaciations during the Eocene and Oligocene.

To more fully resolve glacial and sea ice dynamics, given coring gaps in current sedimentary archives and limitations with existing age models, it is critical to recover new and complete Cenozoic sedimentary successions from the Arctic and Southern Ocean. Continuous successions will also enable the development of high-resolution records that could provide constraints on the role of orbital forcing in governing cryospheric stability. The ability to generate paired records of IRD and carbonate stable isotope ratios in a single core, which is not possible at Site 913 due to the lack of preserved carbonate, may shed light on past changes in the extent of Greenland ice and on the relative timing of glaciation in the Northern Hemisphere compared to fluctuations in global ice budgets during the Cenozoic. An extension of the Arctic source database to include presently undocumented regions, including in Northeastern Greenland, would place more complete constraints on the spatial extent of ice on Greenland in the past, although some sources may have been eroded away. Such research would shed light on the behavior of an ancient Greenland ice cap or ice sheet under warm and polythermal glacial regimes that presumably existed during the middle Eocene through Oligocene. In addition, the application of the IRD source fingerprinting method and other techniques including weathering proxies, to new and existing sediment core sites, will allow us to gain a more complete picture of Greenland glacial ice and Arctic sea ice dynamics under higher CO_2_ regimes.

## Methods

### Overview

To critically test the hypothesis that glacial ice occurred ephemerally on Greenland beginning in the middle Eocene and became more stable after the Eocene–Oligocene transition, we applied an accurate, precise, and well-established geochemical fingerprinting technique that allows us to identify the specific regions of origin for IRD^9–11,37,43^. By characterizing multi-element signatures of detrital iron oxide minerals that occur in core samples, we are able to isolate sources of IRD over time, determine the sources and shed light on the relative extent of glacial ice on Greenland, and decipher contributions of sea ice from specific circum-Arctic sources during a given interval. We constrain Greenland glacial ice and Arctic sea ice dynamics using data from Site 913 during the middle Eocene through early Oligocene interval (47–26 Ma). We also compare our results with published data, including other proxies for sea ice, glacial ice, and ice volume. Our results are compared to extensive carbon cycle proxy data sets that enable us to study the relationship between Northern Hemisphere cryosphere dynamics and atmospheric *p*CO_2_, and to investigate causal mechanisms, such as whether fluctuations in CO_2_ may have driven the transition to a Cenozoic climate characterized by glacial maxima in both hemispheres, i.e., episodic bipolar glaciations.

Results were compared with published data on IRD, including mass accumulation rates, dropstone occurrence, and Arctic sea ice diatom occurrence^12,13,15,37^. These records are also compared to a global benthic *δ*^18^O and *δ*^13^C synthesis, a seawater *δ*^18^O synthesis, estimates of the carbonate compensation depth, and reconstructed atmospheric *p*CO_2_ from multiple proxies^1,3,22,56–58^. A full list of publications that are the sources of data used for comparison to our results, are listed in the Supplementary Methods.

### Fe grain matching

Iron oxides are dominant accessory minerals in most sediments and rocks and are highly durable. Their diverse geochemistry (e.g., with potential substitutions of several elements for Fe in mineral lattices) gives rise to unique regional chemo-geographic patterns. Therefore the chemistry of individual mineral grains can be used to pinpoint each grain’s region of provenance^9,43^.

Samples were measured and data analyzed blindly at Old Dominion University. The method for precise source determination uses the chemical signature of 14 elements in nine types of iron oxide minerals^37,43^. The anhydrous detrital Fe oxide grains (Fe grains) were separated magnetically using a hand magnet and the Frantz magnetic separator on the sieved 45–250 μm size fractions. These grains were then mounted in epoxy plugs, polished, and photographed so that each Fe grain could be identified as to location on the photo and mineral type for later analysis on the Cameca SX100 electron probe microanalyzer (EPMA). The mineral identifications were checked after EPMA analysis to insure that the microscopic identification done with a reflected light microscope (1000×) were correct. Each grain was analyzed for 14 elements (Fe, Ti, O, Mn, Mg, Si, Al, Ca, Zn, V, Ni, Cr, Nb, and Ta). Each grain was then matched to grains of the same mineral type from the entire Arctic database of over 39,000 Fe grain analyses for all 14 elements for nearshore and onshore samples taken from 284 sample sites^43^. These Fe oxides formed in igneous and metamorphic rocks that almost always predated the Eocene (most are much older) and have been eroded and deposited in Pleistocene and younger deposits where they were sampled for the database. Each element has to be within 2 standard deviations of multiple analyses of hundreds of selected grains with high values of each element.

This technique enables us to trace each grain to a specific source region (Fig. 1). The uncertainty of this method has been quantified, with an error rate of incorrect matches of only 1.5%^37,43^. By characterizing all of the grains in a sediment sample, and by knowing the exact source for each grain, we can determine the multiple regions that contributed IRD present in a sediment sample. When more than one source Fe grain matched to an Fe grain from a sample, the sources were pro-rationed according to how close the analyses were to all 14 elements. This provenance tool has also been compared to the use of lithic grains for source determination in several studies with compatible but far more precise results that are cited in the Supplementary Methods.

Thus, we can ascertain the geographic distribution of glacial ice and sea ice that rafted grains to Site 913. We note that while it is possible that matched Fe oxide grains are from unsampled source areas, the probability of Fe oxide grains having the same chemical fingerprint as grains represented in the source database is low (<0.01)^43^. Sample coverage for Northeastern Greenland between source areas #35 and #46 is limited, although this region is not known to have recently produced many icebergs. Source area #46 consists of an inner shelf core (PS2641) with 147 samples from every few centimeters down to 266 cm. All Fe oxide grains matched to sources north of Fram Strait were removed from this database in order to ensure that only Fe oxide grains from East Greenland are represented.

The complete match of all Fe grains in Site 913 samples to Greenland and circum-Arctic sources can be found in Supplementary Data sets 1–2.

### Age model

The sediments recovered from Site 913 have been dated using a reliable age model based on paleomagnetics and biostratigraphy^8,67^.

### Comparison with published records of Arctic sea ice from ACEX

There are data sets for two different sites used to place constraints on Arctic sea ice as shown in Figs. 3–5, and as discussed in the text: (1) Site 913 (IRD provenance data generated in this study shown on the Site 913 age model) and (2) the ACEX site (full list of citations in Supplementary Methods). For ACEX, we note there are two different age models proposed^68,69^ that yield different ages and place the first appearance of IRD in ACEX a few million years apart, and produce different ice-rafting histories for that site. Therefore we show the results of using each of these age models in Fig. 3, with the different color lines in the bottom panel, as described in the figure caption.

### Comparison with ACEX IRD source matching

Estimates for number of ACEX IRD grains that can be matched to a source region comes from a publication^37^.

### Comparison with records of Arctic sea ice

Estimates for Arctic sea ice onset come from multiple publications. Full list of citations in Supplementary Methods.

### Site 913 IRD MAR

Estimates of IRD mass accumulation rates at Site 913 come from a publication^12^.

### Comparison with records of Antarctic ice

Estimates for Antarctic ice storage come from multiple publications. Full list of citations in Supplementary Methods.

### Composite deep-sea benthic foraminiferal *δ*^18^O and *δ*^13^C

Benthic foraminiferal *δ*^18^O and *δ*^13^C are a compilation from multiple publications. Full list of citations in Supplementary Methods.

### Seawater *δ*^18^O

Reconstructed seawater *δ*^18^O from multiple publications. Full list of citations in Supplementary Methods.

### Carbonate compensation depth

Reconstructed tropical Pacific and equatorial Pacific CCD from multiple publications. Full list of citations in Supplementary Methods.

### *p*CO_2_

Proxy atmospheric CO_2_ synthesis contains data from multiple publications. Full list of citations in Supplementary Methods.

### Data availability

The data sets generated or analyzed during this study are included in Supplementary Data 1–4.

## Electronic supplementary material

Supplementary Information

Description of Additional Supplementary Files

Supplementary Data 1

Supplementary Data 2

Supplementary Data 3

Supplementary Data 4
