# Supplementary material for: Evidence for ephemeral middle Eocene to early Oligocene Greenland glacial ice and pan-Arctic sea ice
Source: Nat Commun. 2018 Mar 12;9:1038. doi: 10.1038/s41467-018-03180-5 (PMC5847593; doi:10.1038/s41467-018-03180-5)
Supplement: Supplementary file 1 — Supplementary Information [file 41467_2018_3180_MOESM1_ESM.pdf]

Supplementary Information

Evidence for ephemeral middle Eocene to early Oligocene Greenland glacial ice and pan-Arctic sea ice

Aradhna Tripati and Dennis Darby

Nature Communications

This file contains:

Supplementary Methods

Supplementary Figure 1: Number of Fe oxide grains from Greenland and Arctic Ocean sources at Site 913 from 48 to 26 Ma compared to proxy indicators of global climate, ice volume, and carbon cycle changes. Same as Fig. 4 in main text with vertical blue lines.

Supplementary Figure 2: Same as Fig. 4 in main text but detailed inset of interval from 48-36 Ma with vertical blue lines.

Supplementary Figure 3: Same as Fig. 4 in main text but detailed inset of interval from 36-26 Ma with vertical blue lines.

Supplementary Figure 4: Same as Fig. 4 in main text but detailed inset of interval from 48-36 Ma.

Supplementary Figure 5: Same as Fig. 4 in main text but detailed inset of interval from 36-26 Ma.

References

Supplementary Methods: Contains full set of references for data sources.

Fe grain matching: Samples were measured and data analyzed blindly at Old Dominion University. The method for precise source determination uses the chemical signature of 14 elements in nine types of iron oxide minerals[1–5]. This provenance tool has also been compared to the use of lithic grains for source determination in several studies with compatible but far more precise results[2,6–9].

Comparison with records of Arctic sea ice: There are datasets for two different sites used to place constraints on Arctic sea ice as shown in Figures 3-5, and as discussed in the text: (1) Site 913 (IRD provenance data generated in this study; Data in Supplementary Data Tables 1-2) and (2) the ACEX site[3,10–23] (Data in Supplementary Data Table 3). We note there are two different age models proposed for ACEX[24,25] that yield different ages that places the first appearance of IRD in ACEX a few million years apart and produce different ice-rafting histories, with one age model[25] producing results that are broadly consistent with the data for Site 913. We show the results of using each of these age models in Figure 3, with the different color lines in the bottom panel, as described in the figure caption; all subsequent figures and the discussion uses the age model for ACEX that best matches the results for Site 913.

Comparison with records of Arctic sea ice*:* Estimates for Arctic sea ice onset come from multiple publications[3,10–23].

Comparison with records of Antarctic ice: Estimates for Antarctic ice storage come from multiple publications[26–37].

Composite deep-sea benthic foraminiferal $\delta^{18}O$ and $\delta^{13}C$: Benthic foraminiferal δ^{18}O and δ^{13}C are a compilation from multiple publications and sources therein[31,33,38,39]. Oxygen isotope adjustments are from publications[30,31,40,41]. Data are in Supplementary Data Table 4.

Seawater $\delta^{18}O$: Seawater δ^{18}O from multiple publications and sources therein[33,38,39,42]. Data are in Supplementary Data Table 4.

Carbonate compensation depth: Reconstructed tropical Pacific and equatorial Pacific CCD from multiple publications[38,43]. Data are in Supplementary Data Table 4.

pCO_2: Proxy atmospheric CO_2 synthesis contains data from multiple publications and sources therein[44–85]. Data are in Supplementary Data Table 4.

Supplementary Figure 1: Number of Fe oxide grains from Greenland and Arctic Ocean sources at Site 913 from 48 to 26 Ma compared to proxy indicators of global climate, ice volume, and carbon cycle changes. Vertical blue lines mark intervals where Greenland-sourced IRD at Site 913 occurs or when there is an increase in $\delta^{18}O$. Comparison shows the occurrence of Greenland ice and circum-Arctic sea ice at Site 913 sometimes, though not always, coincides with increasing benthic foraminiferal $\delta^{18}O$ and Pacific water $\delta^{18}O$, increases in the carbonate compensation depth, and relatively low pCO_2. **A.** Ice-rafted Fe oxide grains from different source regions. Horizontal blue lines indicate 2 and 5 grains matched. **B.** IRD mass accumulation rates (MAR) at Site 913 shown, with intervals containing dropstones or grains >250 um in size indicated by underlying dotted blue line. **C.** Composite deep-sea benthic foraminiferal $\delta^{18}O$ record is shown with a 3-point and 5-point running mean. **D.** Changes in water isotopes at sites in the intermediate and deep Pacific are shown, with 3-point running mean for each, is plotted with a low-resolution global composite of reconstructed water $\delta^{18}O$. Underlying blue dotted lines indicate intervals where reconstructions show an increase in water $\delta^{18}O$ of more than 0.6 ‰. **E.** Carbonate compensation depth (CCD) for equatorial Pacific and tropical Pacific are shown, which are impacted by changes in sea level and carbon cycling. Dotted lines indicating where CCD is relatively deep or increases in depth. **F.** Composite proxy pCO_2 reconstruction is shown with lines indicating minimum and maximum values, and grey dotted line marking 500 ppmv. Complete list of data sources in Supplementary Methods. Same as Figure 4 but with no vertical lines.

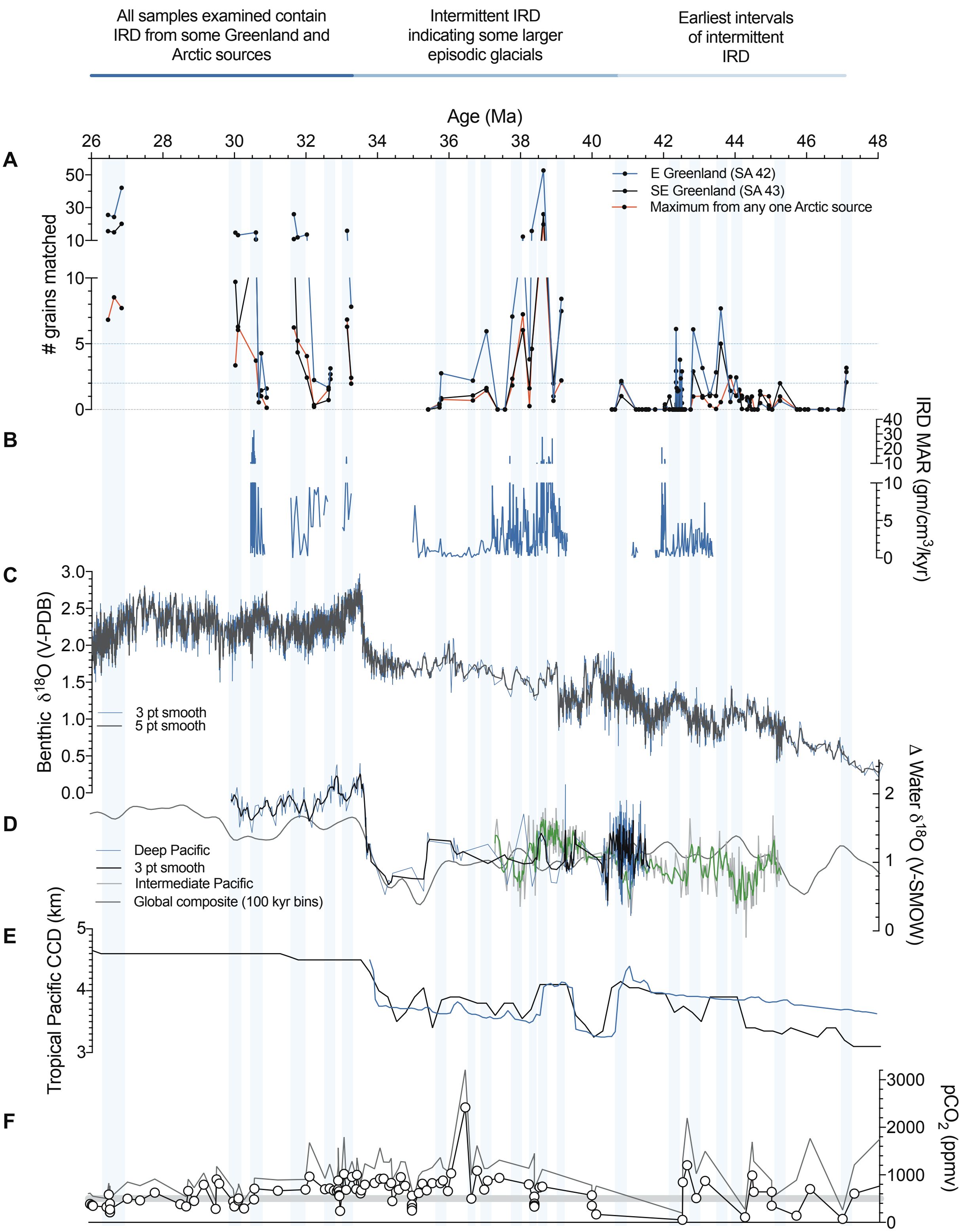

All samples examined contain IRD from some Greenland and Arctic sources
Intermittent IRD indicating some larger episodic glacials
Earliest intervals of intermittent IRD
Age (Ma)
A
B
C
D
E
F
grains matched
E Greenland (SA 42)
SE Greenland (SA 43)
Maximum from any one Arctic source
IRD MAR (gm/cm³/kyr)
Benthic δ18O (V-PDB)
3 pt smooth
5 pt smooth
Deep Pacific
3 pt smooth
Intermediate Pacific
Global composite (100 kyr bins)
Δ Water δ18O (V-SMOW)
Tropical Pacific CCD (km)
pCO2 (ppmv)

Supplementary Figure 2: Number of Fe oxide grains from Greenland and Arctic Ocean sources at Site 913 from 48 to 36 Ma compared to proxy indicators of global climate, ice volume, and carbon cycle changes. Vertical blue lines mark intervals where Greenland-sourced IRD at Site 913 occurs or when there is an increase in $\delta^{18}O$. Comparison shows the occurrence of Greenland ice and circum-Arctic sea ice at Site 913 sometimes, though not always, coincides with increasing benthic foraminiferal $\delta^{18}O$ and Pacific water $\delta^{18}O$, increases in the carbonate compensation depth, and relatively low pCO_2. **A.** Ice-rafted Fe oxide grains from different source regions. Horizontal blue lines indicate 2 and 5 grains matched. **B.** IRD mass accumulation rates (MAR) at Site 913 shown, with intervals containing dropstones or grains >250 um in size indicated by underlying dotted blue line. **C.** Composite deep-sea benthic foraminiferal $\delta^{18}O$ record is shown with a 3-point and 5-point running mean. **D.** Changes in water isotopes at sites in the intermediate and deep Pacific are shown, with 3-point running mean for each, is plotted with a low-resolution global composite of reconstructed water $\delta^{18}O$. Underlying blue dotted lines indicate intervals where reconstructions show an increase in water $\delta^{18}O$ of more than 0.6 ‰. **E.** Carbonate compensation depth (CCD) for equatorial Pacific and tropical Pacific are shown, which are impacted by changes in sea level and carbon cycling. Dotted lines indicating where CCD is relatively deep or increases in depth. **F.** Composite proxy pCO_2 reconstruction is shown with lines indicating minimum and maximum values, and grey dotted line marking 500 ppmv. Complete list of data sources in Supplementary Methods. Same as Fig. 4 in main text but detailed inset of interval from 48-36 Ma and with vertical blue lines.

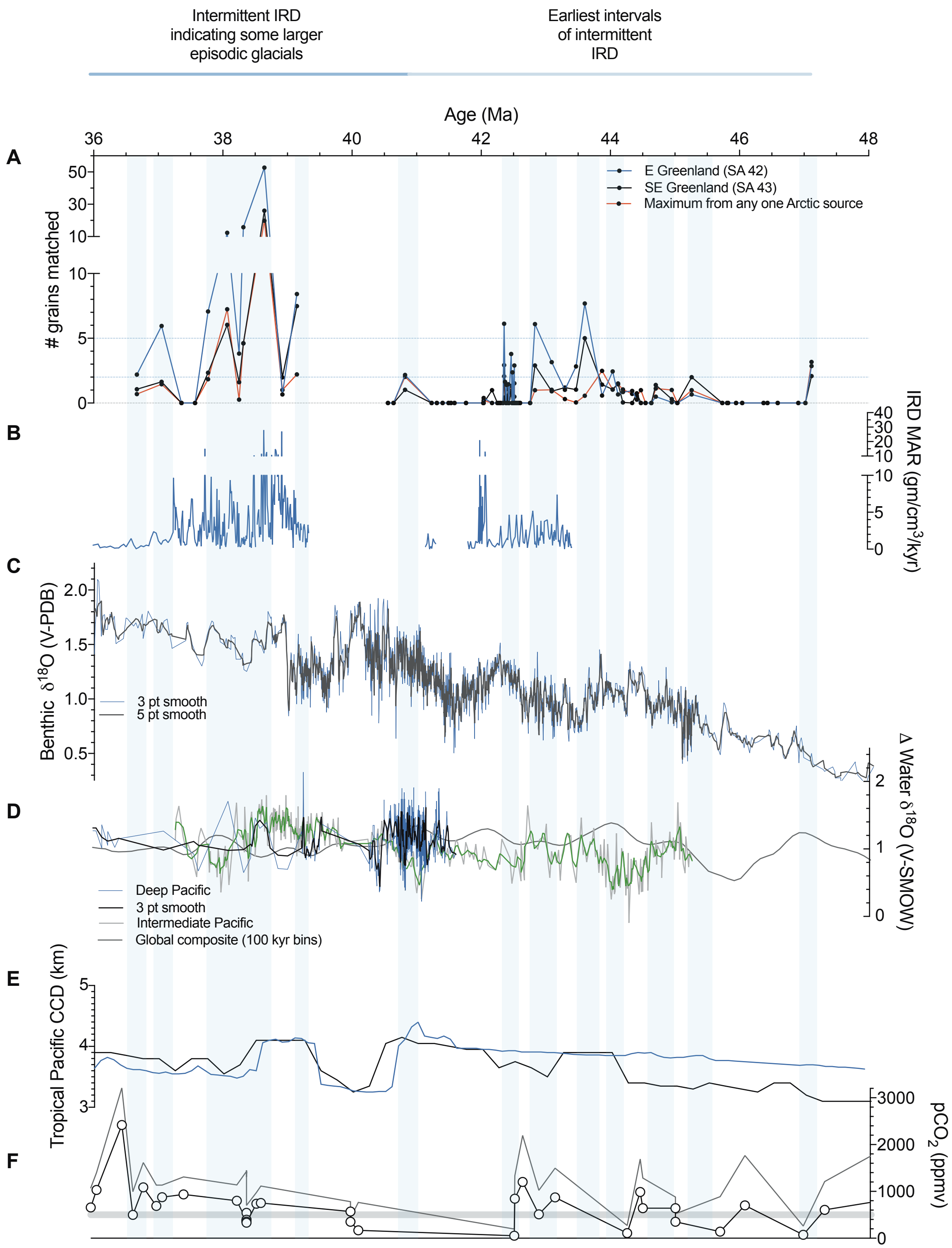

Intermittent IRD indicating some larger episodic glacials
Earliest intervals of intermittent IRD
Age (Ma)
36
38
40
42
44
46
48
A
grains matched
E Greenland (SA 42)
SE Greenland (SA 43)
Maximum from any one Arctic source
B
IRD MAR (gm/cm^3/kyr)
C
Benthic δ^{18}O (V-PDB)
3 pt smooth
5 pt smooth
D
Δ Water δ^{18}O (V-SMOW)
Deep Pacific
3 pt smooth
Intermediate Pacific
Global composite (100 kyr bins)
E
Tropical Pacific CCD (km)
F
pCO$_2$ (ppmv)

Supplementary Figure 3: Number of Fe oxide grains from Greenland and Arctic Ocean sources at Site 913 from 36 to 26 Ma compared to proxy indicators of global climate, ice volume, and carbon cycle changes. Vertical blue lines mark intervals where Greenland-sourced IRD at Site 913 occurs or when there is an increase in $\delta^{18}O$. Comparison shows the occurrence of Greenland ice and circum-Arctic sea ice at Site 913 sometimes, though not always, coincides with increasing benthic foraminiferal $\delta^{18}O$ and Pacific water $\delta^{18}O$, increases in the carbonate compensation depth, and relatively low pCO_2. **A.** Ice-rafted Fe oxide grains from different source regions. Horizontal blue lines indicate 2 and 5 grains matched. **B.** IRD mass accumulation rates (MAR) at Site 913 shown, with intervals containing dropstones or grains >250 um in size indicated by underlying dotted blue line. **C.** Composite deep-sea benthic foraminiferal $\delta^{18}O$ record is shown with a 3-point and 5-point running mean. **D.** Changes in water isotopes at sites in the intermediate and deep Pacific are shown, with 3-point running mean for each, is plotted with a low-resolution global composite of reconstructed water $\delta^{18}O$. Underlying blue dotted lines indicate intervals where reconstructions show an increase in water $\delta^{18}O$ of more than 0.6 ‰. **E.** Carbonate compensation depth (CCD) for equatorial Pacific and tropical Pacific are shown, which are impacted by changes in sea level and carbon cycling. Dotted lines indicating where CCD is relatively deep or increases in depth. **F.** Composite proxy pCO_2 reconstruction is shown with lines indicating minimum and maximum values, and grey dotted line marking 500 ppmv. Complete list of data sources in Supplementary Methods. Same as Fig. 4 in main text but detailed inset of interval from 36-26 Ma and with vertical blue lines.

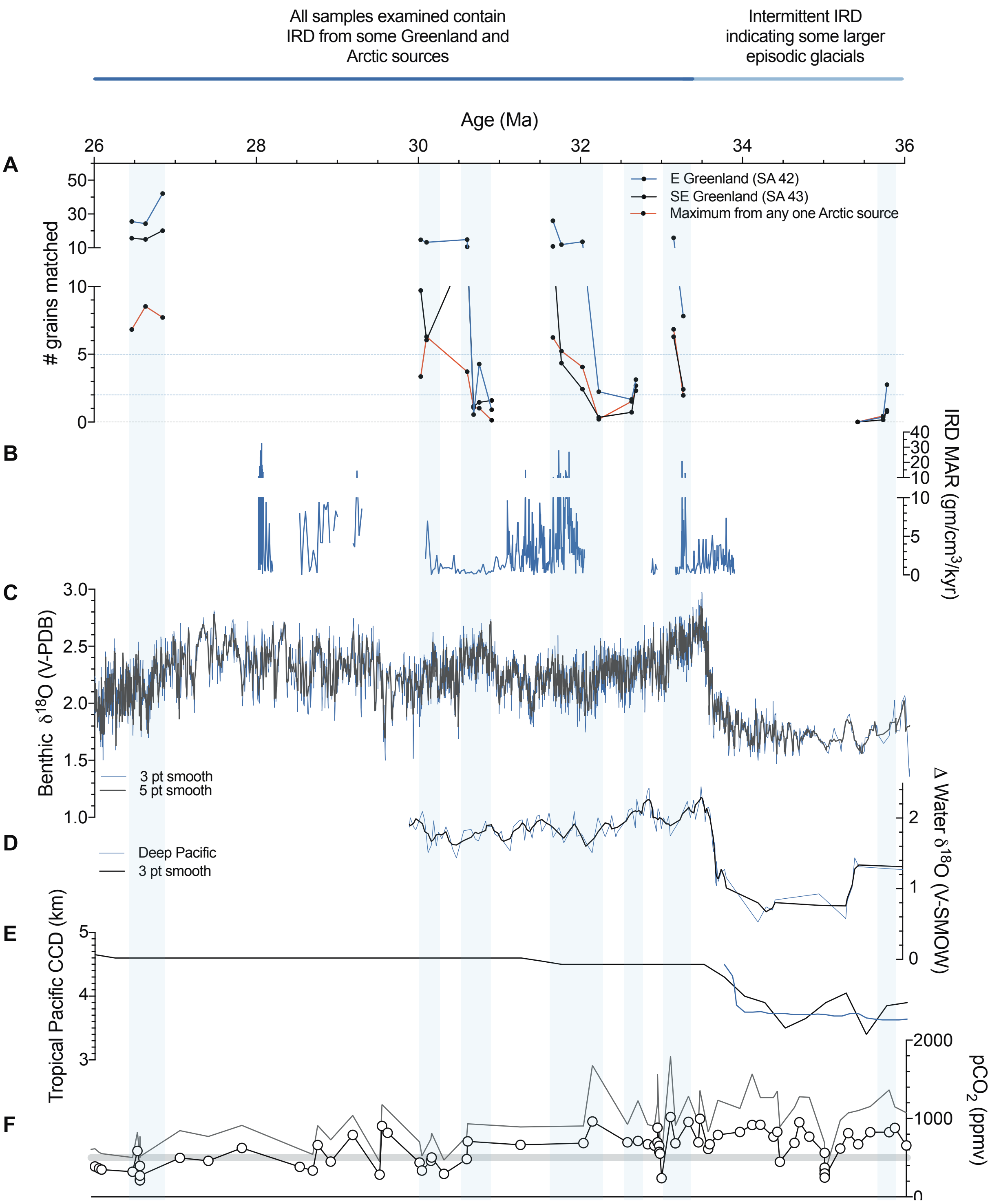
All samples examined contain IRD from some Greenland and Arctic sources
Intermittent IRD indicating some larger episodic glacials
Age (Ma)
26
28
30
32
34
36
A
B
C
D
E
F
grains matched
50
30
10
10
5
0
E Greenland (SA 42)
SE Greenland (SA 43)
Maximum from any one Arctic source
IRD MAR (gm/cm3/kyr)
40
30
20
10
10
5
0
Benthic δ18O (V-PDB)
3.0
2.5
2.0
1.5
1.0
3 pt smooth
5 pt smooth
Δ Water δ18O (V-SMOW)
2
1
0
Deep Pacific
3 pt smooth
Tropical Pacific CCD (km)
5
4
3
pCO2 (ppmv)
2000
1000
0

Supplementary Figure 4: Number of Fe oxide grains from Greenland and Arctic Ocean sources at Site 913 from 48 to 36 Ma compared to proxy indicators of global climate, ice volume, and carbon cycle changes. Comparison shows the occurrence of Greenland ice and circum-Arctic sea ice at Site 913 sometimes, though not always, coincides with increasing benthic foraminiferal $\delta^{18}O$ and Pacific water $\delta^{18}O$, increases in the carbonate compensation depth, and relatively low pCO_2. **A.** Ice-rafted Fe oxide grains from different source regions. Horizontal blue lines indicate 2 and 5 grains matched. **B.** IRD mass accumulation rates (MAR) at Site 913 shown, with intervals containing dropstones or grains >250 um in size indicated by underlying dotted blue line. **C.** Composite deep-sea benthic foraminiferal $\delta^{18}O$ record is shown with a 3-point and 5-point running mean. **D.** Changes in water isotopes at sites in the intermediate and deep Pacific are shown, with 3-point running mean for each, is plotted with a low-resolution global composite of reconstructed water $\delta^{18}O$. Underlying blue dotted lines indicate intervals where reconstructions show an increase in water $\delta^{18}O$ of more than 0.6 ‰. **E.** Carbonate compensation depth (CCD) for equatorial Pacific and tropical Pacific are shown, which are impacted by changes in sea level and carbon cycling. Dotted lines indicating where CCD is relatively deep or increases in depth. **F.** Composite proxy pCO_2 reconstruction is shown with lines indicating minimum and maximum values, and grey dotted line marking 500 ppmv. Complete list of data sources in Supplementary Methods. Same as Fig. 4 in main text but detailed inset of interval from 48-36 Ma.

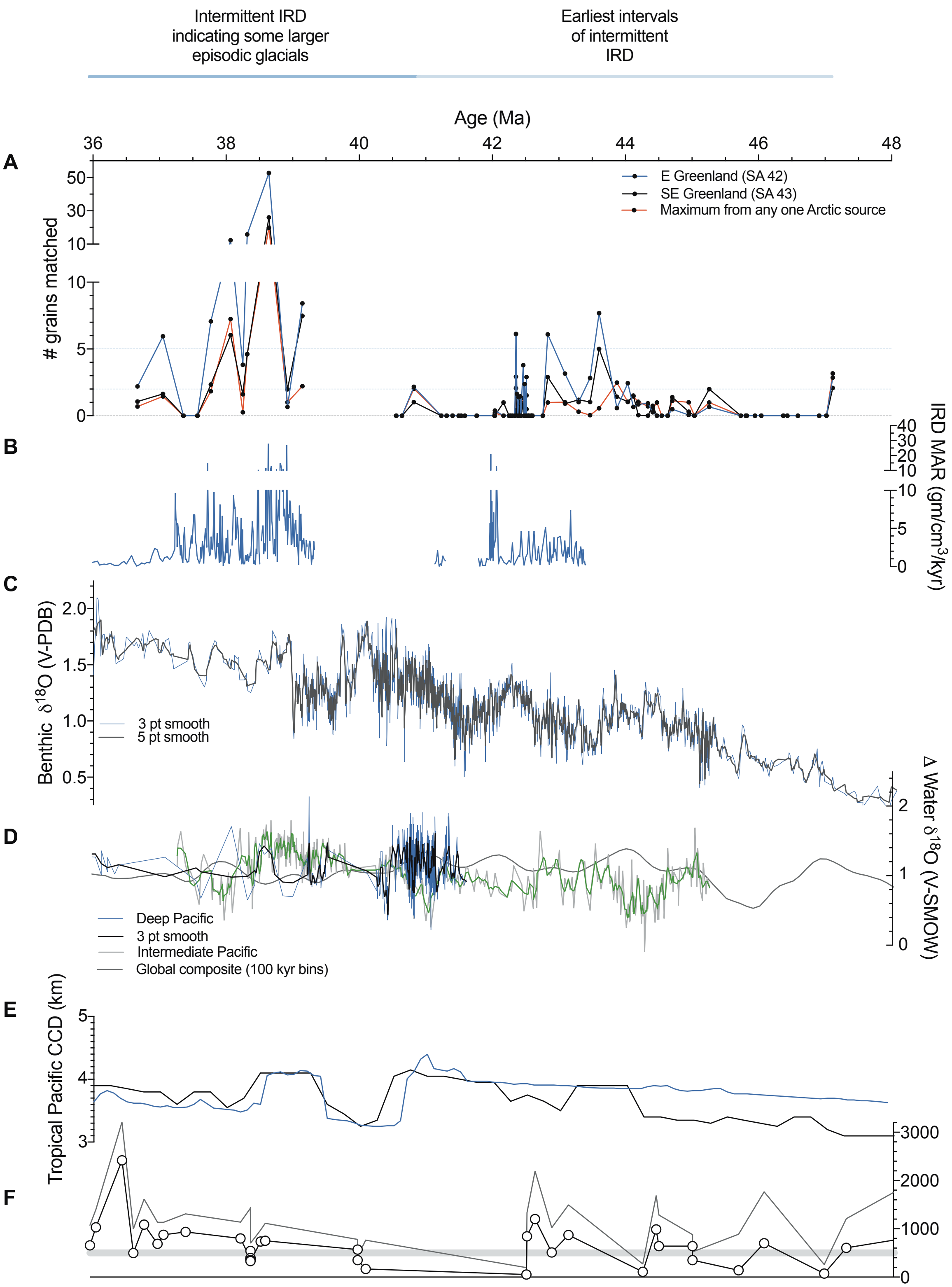

Intermittent IRD indicating some larger episodic glacials
Earliest intervals of intermittent IRD
Age (Ma)
36
38
40
42
44
46
48
A
grains matched
E Greenland (SA 42)
SE Greenland (SA 43)
Maximum from any one Arctic source
B
IRD MAR (gm/cm3/kyr)
C
Benthic δ18O (V-PDB)
3 pt smooth
5 pt smooth
D
Δ Water δ18O (V-SMOW)
Deep Pacific
3 pt smooth
Intermediate Pacific
Global composite (100 kyr bins)
E
Tropical Pacific CCD (km)
F
pCO2 (ppmv)

156
157
158

Supplementary Figure 5: Number of Fe oxide grains from Greenland and Arctic Ocean sources at Site 913 from 36 to 26 Ma compared to proxy indicators of global climate, ice volume, and carbon cycle changes. Vertical blue lines mark intervals where Greenland-sourced IRD at Site 913 occurs or when there is an increase in $\delta^{18}O$. Comparison shows the occurrence of Greenland ice and circum-Arctic sea ice at Site 913 sometimes, though not always, coincides with increasing benthic foraminiferal $\delta^{18}O$ and Pacific water $\delta^{18}O$, increases in the carbonate compensation depth, and relatively low pCO_2. **A.** Ice-rafted Fe oxide grains from different source regions. Horizontal blue lines indicate 2 and 5 grains matched. **B.** IRD mass accumulation rates (MAR) at Site 913 shown, with intervals containing dropstones or grains >250 um in size indicated by underlying dotted blue line. **C.** Composite deep-sea benthic foraminiferal $\delta^{18}O$ record is shown with a 3-point and 5-point running mean. **D.** Changes in water isotopes at sites in the intermediate and deep Pacific are shown, with 3-point running mean for each, is plotted with a low-resolution global composite of reconstructed water $\delta^{18}O$. Underlying blue dotted lines indicate intervals where reconstructions show an increase in water $\delta^{18}O$ of more than 0.6 ‰. **E.** Carbonate compensation depth (CCD) for equatorial Pacific and tropical Pacific are shown, which are impacted by changes in sea level and carbon cycling. Dotted lines indicating where CCD is relatively deep or increases in depth. **F.** Composite proxy pCO_2 reconstruction is shown with lines indicating minimum and maximum values, and grey dotted line marking 500 ppmv. Complete list of data sources in Supplementary Methods. Same as Fig. 4 in main text but detailed inset of interval from 36-26 Ma.

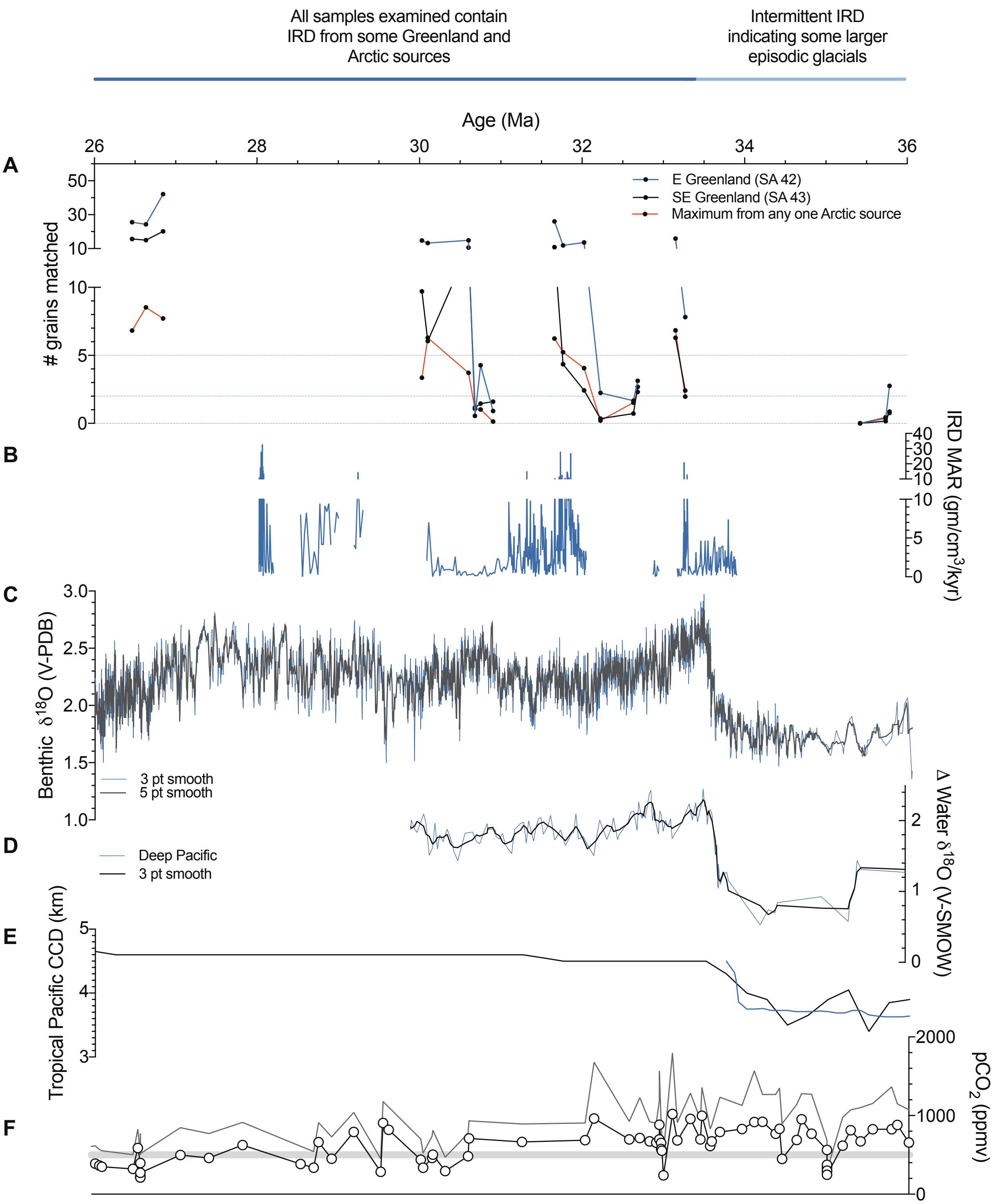
All samples examined contain IRD from some Greenland and Arctic sources
Intermittent IRD indicating some larger episodic glacials
Age (Ma)
26
28
30
32
34
36
A
grains matched
E Greenland (SA 42)
SE Greenland (SA 43)
Maximum from any one Arctic source
B
IRD MAR (gm/cm3/kyr)
C
Benthic δ18O (V-PDB)
3 pt smooth
5 pt smooth
D
Deep Pacific
3 pt smooth
Δ Water δ18O (V-SMOW)
E
Tropical Pacific CCD (km)
F
pCO2 (ppmv)

References

1. Darby, D. A., Myers, W., Herman, S. & Nicholson, B. Chemical Fingerprinting, A Precise and Efficient Method To Determine Sediment Sources. *J. Sediment. Res.* **85,** 247–253 (2015).
2. Bischof, J. F. & Darby, D. A. Mid- to late Pleistocene ice drift in the Western Arctic Ocean: Evidence for a different circulation in the past. *Science* **277,** 74–77 (1997).
3. Darby, D. A. Ephemeral formation of perennial sea ice in the Arctic Ocean during the middle Eocene. *Nat. Geosci.* **7,** 210–213 (2014).
4. Darby, D. A. Sources of sediment found in sea ice from the western Arctic Ocean, new insights into processes of entrainment and drift patterns. *J. Geophys. Res. Oceans* **108,** 3257 (2003).
5. Darby, D. A., Ortiz, J. D., Grosch, C. E. & Lund, S. P. 1,500-year cycle in the Arctic Oscillation identified in Holocene Arctic sea-ice drift. *Nat. Geosci.* **5,** 897–900 (2012).
6. Darby, D. *et al.* New record shows pronounced changes in Arctic Ocean circulation and climate. *Eos Trans. Am. Geophys. Union* **82,** 601–607 (2001).
7. Darby, D. A. & Bischof, J. F. A Holocene record of changing Arctic Ocean ice drift analogous to the effects of the Arctic Oscillation. *Paleoceanography* **19,** doi: 10.1029/2003PA000961 (2004).
8. Bischof, J. F. & Darby, D. A. Quaternary ice transport in the Canadian Arctic and extent of Late Wisconsinan Glaciation in the Queen Elizabeth Islands. *Can. J. Earth Sci.* **36,** 2007–2022 (1999).
9. Darby, D. A. & Bischof, J. F. A Statistical Approach to Source Determination of Lithic and Fe Oxide Grains: An Example from the Alpha Ridge, Arctic Ocean. *J. Sediment. Res.* **66,** 599-607 (1996).
10. Stickley, C. E. *et al.* Evidence for middle Eocene Arctic sea ice from diatoms and ice-rafted debris. *Nature* **460,** 376–379 (2009).
11. Moran, K. *et al.* The Cenozoic palaeoenvironment of the Arctic Ocean. *Nature* **441,** 601–605 (2006).
12. St. John, K. Cenozoic ice-rafting history of the central Arctic Ocean: Terrigenous sands on the Lomonosov Ridge. *Paleoceanography* **23,** doi:10.1029/2007PA001483 (2008).
13. Barron, J. A., Stickley, C. E. & Bukry, D. Paleoceanographic, and paleoclimatic constraints on the global Eocene diatom and silicoflagellate record. *Palaeogeogr. Palaeoclimatol. Palaeoecol.* **422,** 85–100 (2015).
14. Stickley, C. E. *et al.* Variability in the length of the sea ice season in the Middle Eocene Arctic. *Geology* **40,** 727–730 (2012).
15. Immonen, N. Surface microtextures of ice-rafted quartz grains revealing glacial ice in the Cenozoic Arctic. *Palaeogeogr. Palaeoclimatol. Palaeoecol.* **374,** 293–302 (2013).
16. De Schepper, S., Gibbard, P. L., Salzmann, U. & Ehlers, J. A global synthesis of the marine and terrestrial evidence for glaciation during the Pliocene Epoch. *Earth-Sci. Rev.* **135,** 83–102 (2014).
17. Eldrett, J. S., Harding, I. C., Wilson, P. A., Butler, E. & Roberts, A. P. Continental ice in Greenland during the Eocene and Oligocene. *Nature* doi:10.1038/nature05591 (2007).
18. Winkler, A., Wolf-Welling, T., Stattegger, K. & Thiede, J. Clay mineral sedimentation in high northern latitude deep-sea basins since the Middle Miocene (ODP Leg 151, NAAG). *Int. J. Earth Sci.* **91,** 133–148 (2002).

19. Wolf-Welling, T. C. W., Cremer, M., O'Connell, S., Winkler, A. & Thiede, J. Cenozoic Arctic Gateway Paleoclimate Variability: Indications from Changes in Coarse-Fraction Composition. in *Proceedings of Ocean Drilling Program, Scientific Results* (eds. Thiede, J., Myhre, A. M., Firth, J. V., Johnson, G. L. & Ruddiman, W. F.) 515–568 (Ocean Drilling Program, College Station, TX, 1996).
20. St. John, K. E. K. Site 918 IRD mass accumulation rate record, late Miocene-Pleistocene. *Proc. Ocean Drill. Program Sci. Results* **163,** 163–166 (1999).
21. St. John, K. E. K. & Krissek, L. A. Regional patterns of Pleistocene ice-rafted debris flux in the North Pacific. *Paleoceanography* **14,** 653–662 (1999).
22. Polyak, L. *et al.* History of sea ice in the Arctic. *Quat. Sci. Rev.* **29,** 1757–1778 (2010).
23. Helland, P. E. & Holmes, M. A. Surface textural analysis of quartz sand grains from ODP Site 918 off the southeast coast of Greenland suggests glaciation of southern Greenland at 11 Ma. *Palaeogeog Palaeoclim Palaeoecol* **135,** 109–121 (1997).
24. Backman, J. *et al.* Age model and core-seismic integration for the Cenozoic Arctic Coring Expedition sediments from the Lomonosov Ridge. *Paleoceanography* **23,** (2008).
25. Poirier, A. & Hillaire-Marcel, C. Improved Os-isotope stratigraphy of the Arctic Ocean. *Geophys. Res. Lett.* **38,** (2011).
26. Ehrmann, W. U. *et al.* History of Antarctic glaciation: An Indian Ocean Perspective. in *Synthesis of results from scientific Drilling in the Indian Ocean* (eds. Duncan, R. A. & Rea, D.) 423–446 (Amer. Geophys. Union, Washington, D.C., 1992).
27. Ehrmann, W. U. & Mackensen, A. Sedimentologic evidence for the formation of an East Antarctic ice sheet in Eocene/Oligocene time. *Palaeogeogr Palaeoclim. Palaeoecol* **93,** 85–112 (1992).
28. Mackensen, A. & Ehrmann, W. U. Middle Eocene through Early Oligocene climate history and paleoceanography in the Southern Ocean: Stable oxygen and carbon isotopes from ODP sites on Maud Rise and Kerguelen Plateau. *Mar. Geol.* **108,** 1–28 (1992).
29. Zachos, J. C., Stott, L. D. & Lohmann, K. C. Evolution of early Cenozoic marine temperatures. *Paleoceanography* **9,** 353–387 (1994).
30. Zachos, J. C., Pagani, M., Sloan, L. C., Thomas, E. & Billups, K. Trends, rhythms, and aberrations in global climate 65 Ma to present. *Science* **292,** 686–693 (2001).
31. Zachos, J., Dickens, G. R. & Zeebe, R. E. An early Cenozoic perspective on greenhouse warming and carbon cycle dynamics. *Nature* **451,** 279–283 (2008).
32. Miller, K. G. *et al.* The Phanerozoic Record of Global Sea-Level Change. *Science* **310,** 1293–1298 (2005).
33. Dawber, C. F. & Tripati, A. K. Constraints on glaciation in the middle Eocene (46–37 Ma) from Ocean Drilling Program (ODP) Site 1209 in the tropical Pacific Ocean. *Paleoceanography* **26,** 10.1029/2010PA002037 (2011).
34. Bohaty, S. & Zachos, J. Significant Southern Ocean warming event in the late middle Eocene. *Geology* **31,** 1017–1020 (2003).
35. Peters, S. E., Carlson, A. E., Kelly, D. C. & Gingerich, P. D. Large-scale glaciation and deglaciation of Antarctica during the Late Eocene. *Geology* **38,** 723–726 (2010).
36. Browning, J., Miller, K. & Pak, D. Global implications of Eocene Greenhouse and Doubthouse sequences on the New Jersey coastal plain; the Icehouse cometh. *Geology* **24,** 639–642 (1996).

37. Pekar, S. F., Hucks, A., Fuller, M. & Li, S. Glacioeustatic changes in the early and middle Eocene (51-42 Ma): Shallow-water stratigraphy from ODP Leg 189 1171 (South Tasman Rise) and deep-sea $\delta^{18}O$ records. *GSA Bull.* **117,** 1081–1093 (2005).
38. Tripati, A. K. Eocene bipolar glaciation associated with global carbon cycle changes. *Nature* **436,** 341–346 (2005).
39. Tripati, A. K. *et al.* Evidence for synchronous glaciation of Antarctica and the Northern Hemisphere during the Eocene and Oligocene: Insights from Pacific records of the oxygen isotopic composition of seawater: *in*. (Antarctica: A Keystone in a Changing World—Online Proceedings for 10th ISAES, edited by AK Cooper and CR Raymond et al., USGS Open-File Report, Reston, VA, 2007).
40. Shackleton, N. J. & Opdyke, N. D. Oxygen Isotope and Palaeomagnetic Stratigraphy of Equatorial Pacific Core V28-238: Oxygen Isotope Temperatures and Ice Volumes on a 10^5 Year and 10^6 Year Scale. *Quat. Res.* **3,** 39–55 (1973).
41. Shackleton, N. J., Hall, M. A. & Boersma, A. Oxygen and carbon isotope data from Leg 74 foraminifers. *Initial Rep. Deep Sea Drill. Proj.* **74,** 599–612 (1984).
42. Cramer, B. S., Miller, K. G., Barrett, P. J. & Wright, J. D. Late Cretaceous–Neogene trends in deep ocean temperature and continental ice volume: Reconciling records of benthic foraminiferal geochemistry ($\delta^{18}O$ and Mg/Ca) with sea level history. *J. Geophys. Res. Oceans* **116,** 10.1029/2011JC007255 (2011).
43. Pälike, H. *et al.* A Cenozoic record of the equatorial Pacific carbonate compensation depth. *Nature* **488,** 609–614 (2012).
44. Beerling, D. J. & Royer, D. L. Convergent Cenozoic CO_2 history. *Nat. Geosci.* **4,** 418–420 (2011).
45. Bai, Y.-J. *et al.* Reconstructing atmospheric CO_2 during the Plio–Pleistocene transition by fossil Typha. *Glob. Change Biol.* **21,** 874–881 (2015).
46. Beerling, D. J., Fox, A. & Anderson, C. W. Quantitative uncertainty analyses of ancient atmospheric CO_2 estimates from fossil leaves. *Am. J. Sci.* **309,** 775–787 (2009).
47. Cerling, T. E. Use of carbon isotopes in paleosols as an indicator of the $P(CO_2)$ of the paleoatmosphere. *Glob Biogeochem Cycles* **6,** 307–314 (1992).
48. Demicco, R. V., Lowenstein, T. K. & Hardie, L. A. Atmospheric pCO_2 since 60 Ma from records of seawater pH, calcium, and primary carbonate mineralogy. *Geology* **31,** 793–796 (2003).
49. Doria, G. *et al.* Declining atmospheric CO_2 during the late Middle Eocene climate transition. *Am. J. Sci.* **311,** 63–75 (2011).
50. Ekart, D. D. A 400 million year carbon isotope record of pedogenic carbonate: implications for paleoatmospheric carbon dioxide. *Am. J. Sci.* **299,** 805–827 (1999).
51. Fletcher, B. J., Brentnall, S. J., Anderson, C. W., Berner, R. A. & Beerling, D. J. Atmospheric carbon dioxide linked with Mesozoic and early Cenozoic climate change. *Nat. Geosci.* **1,** 43–48 (2008).
52. Freeman, K. H. & Hayes, J. M. Fractionation of carbon isotopes by phytoplankton and estimates of ancient CO_2 levels. *Glob. Biogeochem. Cycles* **6,** 185–198 (1992).
53. Franks, P. J. *et al.* New constraints on atmospheric CO_2 concentration for the Phanerozoic. *Geophys. Res. Lett.* **41,** 4685–4694 (2014).
54. Greenop, R., Foster, G. L., Wilson, P. A. & Lear, C. H. Middle Miocene climate instability associated with high-amplitude CO_2 variability. *Paleoceanography* **29,** 845–853 (2014).

55. Greenwood, D. R., Scarr, M. J. & Christophel, D. C. Leaf stomatal frequency in the Australian tropical rainforest tree *Neolitsea dealbata* (Lauraceae) as a proxy measure of atmospheric pCO_2. *Palaeogeogr. Palaeoclimatol. Palaeoecol.* **196,** 375–393 (2003).
56. Grein, M. *et al.* Atmospheric CO_2 from the late Oligocene to early Miocene based on photosynthesis data and fossil leaf characteristics. *Palaeogeogr. Palaeoclimatol. Palaeoecol.* **374,** 41–51 (2013).
57. Henderiks, J. & Pagani, M. Coccolithophore cell size and the Paleogene decline in atmospheric CO_2. *Earth Planet. Sci. Lett.* **269,** 576–584 (2008).
58. Hu, J.-J. *et al.* A new positive relationship between pCO_2 and stomatal frequency in *Quercus guyavifolia* (Fagaceae): a potential proxy for palaeo-CO_2 levels. *Ann. Bot.* **115**, 777-788 (2015).
59. Huang, C., Retallack, G. J., Wang, C. & Huang, Q. Paleoatmospheric pCO_2 fluctuations across the Cretaceous–Tertiary boundary recorded from paleosol carbonates in NE China. *Palaeogeogr. Palaeoclimatol. Palaeoecol.* **385,** 95–105 (2013).
60. Hyland, E. G. & Sheldon, N. D. Coupled CO_2-climate response during the Early Eocene Climatic Optimum. *Palaeogeogr. Palaeoclimatol. Palaeoecol.* **369,** 125–135 (2013).
61. Koch, P. L., Zachos, J. C. & Gingerich, P. D. Correlation between isotope records in marine and continental carbon reservoirs near the Palaeocene/Eocene boundary. *Nature* **358,** 319–322 (1992).
62. Kürschner, W. M., van der Burgh, J., Visscher, H. & Dilcher, D. L. Oak leaves as biosensors of late Neogene and early Pleistocene paleoatmospheric CO_2 concentrations. *Mar. Micropaleont.* **27,** 299–312 (1996).
63. Kürschner, W. M., Kvaček, Z. & Dilcher, D. L. The impact of Miocene atmospheric carbon dioxide fluctuations on climate and the evolution of terrestrial ecosystems. *Proc. Natl. Acad. Sci.* **105,** 449–453 (2008).
64. Kürschner, W. M., Wagner, F., Dilcher, D. L. & Visscher, H. Using fossil leaves for the reconstruction of Cenozoic paleoatmospheric CO_2 concentrations. *Geol. Perspect. Glob. Clim. Change APPG Stud. Geol.* **47,** 169–189 (2001).
65. Liu, X. Y., Gao, Q., Han, M. & Jin, J. H. The pCO_2 estimates of the late Eocene in South China based on stomatal density of *Nageia gaertner* leaves. *Climate of the Past Discussions* **11**, 10.5194/cpd-11-2615-2015 (2015).
66. Lowenstein, T. & Demicco, R. Elevated Eocene Atmospheric CO_2 and its Subsequent Decline. *Science* **313,** doi:10.1126/science.1129555 (2006).
67. Martínez-Botí, M. A. *et al.* Plio-Pleistocene climate sensitivity evaluated using high-resolution CO_2 records. *Nature* **518,** 49–54 (2015).
68. Maxbauer, D. P., Royer, D. L. & LePage, B. A. High Arctic forests during the middle Eocene supported by moderate levels of atmospheric CO_2. *Geology* **42,** 1027–1030 (2014).
69. McElwain, J. C. Do fossil plants signal palaeoatmospheric carbon dioxide concentration in the geological past? *Philos. Trans. R. Soc. B Biol. Sci.* **353,** 83–96 (1998).
70. Nordt, L., Atchley, S. & Dworkin, S. I. Paleosol barometer indicates extreme fluctuations in atmospheric CO_2 across the Cretaceous-Tertiary boundary. *Geology* **30,** 703–706 (2002).
71. Pearson, P. N., Foster, G. L. & Wade, B. S. Atmospheric carbon dioxide through the Eocene–Oligocene climate transition. *Nature* **461,** 1110–1113 (2009).
72. Retallack, G. J. Greenhouse crises of the past 300 million years. *Geol. Soc. Am. Bull.* **121,** 1441–1455 (2009).

73. Retallack, G. J. Refining a pedogenic-carbonate CO_2 paleobarometer to quantify a middle Miocene greenhouse spike. *Palaeogeogr. Palaeoclimatol. Palaeoecol.* **281,** 57–65 (2009).
74. Roth-Nebelsick, A. *et al.* Stomatal density and index data of *Platanus neptuni* leaf fossils and their evaluation as a CO_2 proxy for the Oligocene. *Rev. Palaeobot. Palynol.* **206,** 1–9 (2014).
75. Roth-Nebelsick, A., Grein, M., Utescher, T. & Konrad, W. Stomatal pore length change in leaves of *Eotrigonobalanus furcinerv*is (Fagaceae) from the Late Eocene to the Latest Oligocene and its impact on gas exchange and CO_2 reconstruction. *Rev. Palaeobot. Palynol.* **174,** 106–112 (2012).
76. Schubert, B. A. & Jahren, A. H. Reconciliation of marine and terrestrial carbon isotope excursions based on changing atmospheric CO_2 levels. *Nat. Commun.* **4,** 1653 (2013).
77. Seki, O. *et al.* Alkenone and boron-based Pliocene pCO_2 records. *Earth Planet. Sci. Lett.* **292,** 201–211 (2010).
78. Sinha, A. & Stott, L. D. New atmospheric pCO_2 estimates from paleosols during the late Paleocene/early Eocene global warming interval. *Glob. Planet. Change* **9,** 297–307 (1994).
79. Smith, R. Y., Greenwood, D. R. & Basinger, J. F. Estimating paleoatmospheric pCO_2 during the Early Eocene Climatic Optimum from stomatal frequency of Ginkgo, Okanagan Highlands, British Columbia, Canada. *Palaeogeogr. Palaeoclimatol. Palaeoecol.* **293,** 120–131 (2010).
80. Steinthorsdottir, M., Wohlfarth, B., Kylander, M. E., Blaauw, M. & Reimer, P. J. Stomatal proxy record of CO_2 concentrations from the last termination suggests an important role for CO_2 at climate change transitions. *Quat. Sci. Rev.* **68,** 43–58 (2013).
81. Stott, L. D. Higher temperature and lower oceanic pCO_2: A climate enigma at the end of the Paleocene Epoch. *Paleoceanography* **7,** 395–404 (1992).
82. Stults, D. Z., Wagner-Cremer, F. & Axsmith, B. J. Atmospheric paleo-CO_2 estimates based on *Taxodium distichum* (Cupressaceae) fossils from the Miocene and Pliocene of Eastern North America. *Palaeogeogr. Palaeoclimatol. Palaeoecol.* **309,** 327–332 (2011).
83. Tripati, A. K., Roberts, C. D. & Eagle, R. A. Coupling of CO_2 and Ice Sheet Stability Over Major Climate Transitions of the Last 20 Million Years. *Science* doi:10.1126/science.1178296, (2009).
84. Tripati, A. K., Roberts, C. D., Eagle, R. A. & Li, G. A 20 million year record of planktic foraminiferal B/Ca ratios: Systematics and uncertainties in pCO_2 reconstructions. *Geochim. Cosmochim. Acta* **75,** 2582–2610 (2011).
85. Zhang, Y. G., Pagani, M., Liu, Z., Bohaty, S. M. & DeConto, R. A 40-million-year history of atmospheric CO_2. *Philos. Trans. R. Soc. Lond. Math. Phys. Eng. Sci.* **371,** 20130096 (2013).
