# Supplementary material for: Evidence for ephemeral middle Eocene to early Oligocene Greenland glacial ice and pan-Arctic sea ice
Source: Nat Commun. 2018 Mar 12;9:1038. doi: 10.1038/s41467-018-03180-5 (PMC5847593; doi:10.1038/s41467-018-03180-5)
Supplement: Supplementary file 2 — Description of Additional Supplementary Files [file 41467_2018_3180_MOESM2_ESM.pdf]

Description of Additional Supplementary Files

File Name: Supplementary Data 1
Description: New data constraining source area (SA) fractions at Site 913. Number of Fe grains matched to sources for each Fe grain analyzed. NaN = unknown match where all or some of the 14 elements did not meet minimum criteria of two standard deviations based on hundreds of replicate analyses of these elements. Fractional counts sum to one and indicate that more than one source area is possible

File Name: Supplementary Data 2
Description: New data constraining source area (SA) fractions at Site 913 as a function of sample depth and age. Number of Fe grain matches summed for each source area.

File Name: Supplementary Data 3
Description: Published data on # Fe grains matched from sources that would have taken over one year to drift to the ACEX core site. Data sources in Supplemental Methods.

File Name: Supplementary Data 4
Description: Published proxy syntheses of CO_2, $\delta^{18}O$, δ^{13} C, and carbonate compensation depth used in figures. Data sources in Supplemental Methods.
